# What’s in a question-Using patterns in language to correctly identify psychiatric consultation requests

**DOI:** 10.1192/j.eurpsy.2025.1241

**Published:** 2025-08-26

**Authors:** I. E. Leppla, M. Faeder

**Affiliations:** 1 Psychiatry and Behavioral Sciences, Johns Hopkins School of Medicine, Baltimore; 2 Psychiatry, University of Pittsburgh School of Medicine, Pittsburgh, United States

## Abstract

**Introduction:**

In consultation-liaison work, oftentimes the “consult question” is elusive. Primary teams can sense that there is a need for psychiatric consultation, but may not be able to formulate those concerns into the “right question.” Therefore, when the consult team receives the question, the presenting problem may be very different from the reason for consultation. This is never more true than in the case of delirium. Studies have shown that delirium often goes unrecognized by medical and surgical services. (Armstrong et al. Psychosomatics 1997;38:433-439).

**Objectives:**

This study sought to determine the reasons for consultation when a diagnosis of delirium was given by the consulting psychiatrist.

**Methods:**

A retrospective chart review of one year of consecutive consults to the Psychiatry Consult & Liaison service was done for three hospitals comprising the majority of consults to the University of Pittsburgh Medical Center C&L service originating from other teaching services. This yielded approximately 3000 new consults. Each consult was coded for the following variables: the exact quotation from the referring team, the summary statement of what the consultant felt were the most relevant issues, and the DSM-V F-code. We analyzed the consults that were either specific requests for help managing delirium or that resulted in a diagnosis of delirium by the C&L service.

**Results:**

We found that a large majority of consults for “agitation” were ultimately aimed at helping to manage a patient with low cognitive reserve, in the form of dementia, delirium or traumatic brain injury. “AMA” consults were typically involving a patient withdrawing from alcohol or opioids. “Lack of engagement with the treatment team” corresponded to many patients who were delirious. Of the consults that resulted in the diagnosis of delirium the most common reasons for consultation were: delirium/altered mental status (AMS) (21%), depression/adjustment (16%), agitation (11%), psychosis (11%), substance use or overdose (10%), and previously existing psychiatric disorder (26%). Often more than one reason was given for consultation. When only one reason was given previously existing psychiatric disorder (18%), depression (11%), and delirium/AMS (10%) were the most likely reasons for consultation.

**Conclusions:**

The role of the psychiatric team is often to help primary teams figure out which question to ask, and this is then the first step towards getting the patient the help they need. This work adds to the literature because shows patterns between consultation requests and ultimate diagnoses. If we can notice patterns in the diagnoses based on the types of questions that are asked, this may help teach the teams to broaden their differential to include patterns that we have noticed.

**Disclosure of Interest:**

None Declared

